# Genomics and Multi-Omics Perspectives on the Pathogenesis of Cardiorenal Syndrome

**DOI:** 10.3390/genes16111303

**Published:** 2025-11-01

**Authors:** Song Peng Ang, Jia Ee Chia, Eunseuk Lee, Madison Laezzo, Riddhi Machchhar, Sakhi Patel, George Davidson, Vikash Jaiswal, Jose Iglesias

**Affiliations:** 1 Division of Cardiology, Sarver Heart Center, University of Arizona, Tucson, AZ 85724, USA; 2 Department of Medicine, Texas Tech University Health Science Center, El Paso, TX 79905, USA; jchia@ttuhsc.edu; 3 Department of Medicine, Rutgers Health Community Medical Center, Toms River, NJ 08755, USA; eunseuk.lee@rwjbh.org (E.L.); georgeobiidavidson@outlook.com (G.D.); 4 Department of Medicine, Hackensack Meridian School of Medicine, Nutley, NJ 07110, USA; madison.laezzo@hmhn.org; 5 Department of Medicine, Ocean University Medical Center, Brick, NJ 08724, USA; riddhirm@gmail.com (R.M.); sakhipatel14@gmail.com (S.P.); 6 Department of Cardiovascular Medicine, NorthShore University Health System, Evanston, IL 60201, USA; vikash29jaxy@gmail.com

**Keywords:** cardiorenal, genomics, heart failure, multi-omics, chronic kidney disease

## Abstract

Background: Cardiorenal syndrome (CRS) reflects bidirectional heart–kidney injury whose mechanisms extend far beyond hemodynamics. High-throughput genomics and multi-omics now illuminate the molecular circuits that couple cardiac and renal dysfunction. Methods: We narratively synthesize animal and human studies leveraging transcriptomics, proteomics, peptidomics, metabolomics, and non-coding RNA profiling to map convergent pathways in CRS and to highlight biomarker and therapeutic implications. Results: Across acute and chronic CRS models, omics consistently converge on extracellular matrix (ECM) remodeling and fibrosis (e.g., FN1, POSTN, collagens), immune–inflammatory activation (IL-6 axis, macrophage/complement signatures), renin–angiotensin–aldosterone system hyperactivity, oxidative stress, and metabolic/mitochondrial derangements in both organs. Single-nucleus and bulk transcriptomes reveal tubular dedifferentiation after cardiac arrest-induced AKI and myocardial reprogramming with early CKD, while quantitative renal proteomics in heart failure demonstrates marked upregulation of ACE/Ang II and pro-fibrotic matricellular proteins despite near-normal filtration. Human translational data corroborate these signals: urinary peptidomics detects CRS-specific collagen fragments and protease activity, and circulating FN1/POSTN and selected microRNAs (notably miR-21) show diagnostic potential. Epigenetic and microRNA networks appear to integrate these axes, nominating targets such as anti-miR-21 and anti-fibrotic strategies; pathway-directed repurposing exemplifies dual-organ benefit. Conclusions: Genomics and multi-omics recast CRS as a systems disease driven by intertwined fibrosis, inflammation, neurohormonal and metabolic programs. We propose a translational framework that advances (i) composite biomarker panels combining injury, fibrosis, and regulatory RNAs; (ii) precision, pathway-guided therapies; and (iii) integrated, longitudinal multi-omics of well-phenotyped CRS cohorts to enable prediction and personalized intervention.

## 1. Introduction

Cardiorenal syndrome (CRS) refers to the bidirectional dysfunction of the heart and kidneys, where acute or chronic impairment of one organ precipitates dysfunction in the other [1,2]. A consensus classification by Ronco et al. divides CRS into five subtypes: Type 1 (acute cardio-renal) is acute heart failure leading to acute kidney injury (AKI); Type 2 (chronic cardio-renal) is chronic heart failure leading to chronic kidney disease (CKD); conversely, Type 3 (acute reno-cardiac) is AKI causing acute cardiac decompensation; Type 4 is CKD contributing to chronic cardiac dysfunction; and Type 5 (secondary CRS) involves systemic conditions (e.g., sepsis) simultaneously damaging heart and kidney [1]. This classification underscores the diverse pathophysiological trajectories, although in practice the boundaries often blur. Epidemiologically, heart–kidney overlap is common. For instance, roughly 45–63% of chronic heart failure patients have concomitant CKD, highlighting CRS as a prevalent clinical entity [3].

Historically, CRS pathogenesis was attributed largely to hemodynamic interactions: depressed cardiac output was thought to reduce renal perfusion (lower glomerular filtration) and trigger fluid retention, thereby worsening cardiac load [2,4,5]. However, clinical observations challenged this simple model—improving cardiac function in acute and chronic heart failure does not reliably reverse renal decline, implying additional mechanisms at play [5]. Indeed, current evidence paints CRS as a multifactorial disorder involving neurohormonal activation, inflammation, and molecular crosstalk beyond pure hemodynamics [3,6]. Proposed contributors include endothelial injury, immune dysregulation, fibrosis, oxidative stress, apoptosis, and activation of the renin–angiotensin–aldosterone system (RAAS), among others [3,7]. These intertwined pathways create a vicious cycle of organ cross-talk that remains only partially understood. In recent years, high-throughput “omics” technologies, including genomics, transcriptomics, epigenomics, proteomics and metabolomics have emerged as powerful tools to dissect complex various disorders [8]. By offering a genetic and systems-level perspective, multi-omics studies are uncovering novel mediators of heart–kidney interactions that were previously unrecognized with traditional [7]. In this review, we discuss experimental models and translational omics data that shed light on genetic and molecular mechanisms of CRS, survey key gene activation patterns in various CRS subtypes, and explore the implications of these findings for diagnosis and therapy. We also highlight current limitations and the need for integrative multi-omics strategies to fully elucidate the cardio-renal crosstalk.

## 2. Experimental Models and Omics Insights

### Animal Models of CRS and Transcriptomic Profiling

**Figure 1:**
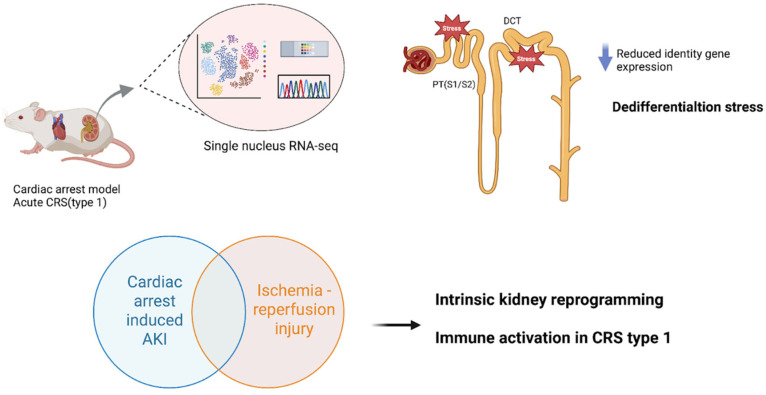
Acute CRS (Type 1): Cardiac Arrest and Resuscitation Model. Mouse models of cardiac arrest and resuscitation have been used to study acute cardiorenal syndrome (CRS), where abrupt heart failure leads to acute kidney injury (AKI). Single-nucleus RNA sequencing (RNA-seq) of the kidney reveals significant transcriptomic changes, especially in proximal tubules (PT, segments S1/S2) and distal convoluted tubules (DCT), which exhibit loss of identity gene expression and dedifferentiation stress. Importantly, the renal transcriptomic profile after cardiac arrest-induced AKI closely mirrors that of human AKI biopsy samples, underscoring translational relevance. Gene enrichment analyses also implicate immune-inflammatory pathways, with both overlaps and distinctions compared to ischemia–reperfusion injury. Abbreviations: CRS, cardiorenal syndrome; AKI, acute kidney injury; PT, proximal tubule; S1/S2, proximal tubule segments; DCT, distal convoluted tubule; RNA-seq, RNA sequencing; Kidney snRNA-seq after cardiac arrest/resuscitation (GEO: GSE271437).

Preclinical models have been instrumental in mimicking CRS subtypes and enabling tissue-level “omics” analyses under controlled conditions. In acute CRS (Type 1), where abrupt heart failure causes AKI, a recently developed mouse model of cardiac arrest and resuscitation provides novel insight. Using this acute cardiorenal injury model, single-nucleus RNA sequencing of the kidney revealed significant transcriptomic changes, especially in proximal tubules, which showed reduced expression of key identity genes and signs of dedifferentiation [9]. Injury responses varied across nephron segments, with proximal segments (S1/S2) and distal convoluted tubules exhibiting the greatest dedifferentiation stress [9]. Notably, the renal gene expression profile after cardiac arrest-induced AKI closely mirrored that seen in human AKI biopsy samples, emphasizing the translational relevance of this model. These findings suggest that acute cardiac failure triggers intrinsic kidney cellular reprogramming, through loss of differentiated function and activation of injury-repair programs, beyond what is seen in kidney-specific insults. They also implicate immune-inflammatory pathways in acute CRS, as gene enrichment analyses showed overlaps and differences in immune gene activation between cardiac-induced AKI and a purely renal ischemia–reperfusion injury model. Together, such acute models illuminate how sudden cardiac dysfunction is associated with molecular injury cascades in the kidney that parallel human AKI, providing targets for early intervention in CRS Type 1 (Figure 1).

**Figure 2:**
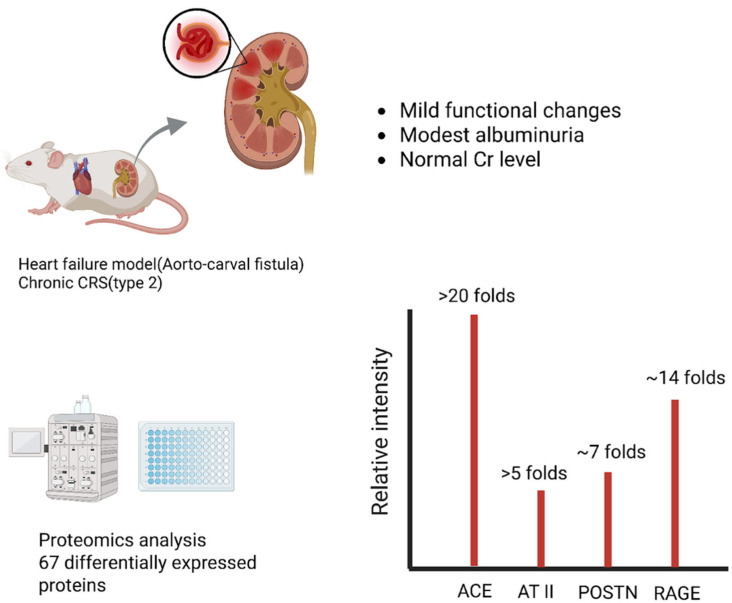
Chronic CRS (Type 2): Heart Failure-Driven Renal Proteome Remodeling. In a rat model of chronic heart failure induced by volume overload (aorto-caval fistula), quantitative proteomics identified 67 differentially expressed renal proteins despite near-normal renal function. Among the most upregulated were angiotensin-converting enzyme (ACE, >20-fold increase) and intrarenal angiotensin II (AT II, >5-fold increase), indicating strong activation of the renin–angiotensin system. Periostin (POSTN), a matricellular protein linked to fibrosis, and the receptor for advanced glycation end-products (RAGE) were also markedly elevated. These findings highlight that subclinical renal injury in chronic CRS is driven by early fibrotic and pro-inflammatory pathways. Abbreviations: CRS, cardiorenal syndrome; ACE, angiotensin-converting enzyme; AT II, angiotensin II; POSTN, periostin; RAGE, receptor for advanced glycation end-products; Renal proteomics in rat ACF model (PRIDE: PXD009296).

Chronic cardio-renal syndrome (Type 2), wherein long-standing heart failure leads to progressive renal impairment, has been studied in animal models of heart failure [10]. In a rat model of chronic heart failure induced by volume overload (aorto-caval fistula), investigators performed quantitative proteomics on kidney tissue to identify pathways of “subclinical” renal injury [11]. After 21 weeks of heart failure, the rats showed reduced renal perfusion and mild functional changes (e.g., modest albuminuria) despite near-normal serum creatinine [11]. Proteomic analysis, however, revealed 67 differentially expressed renal proteins (≥1.5-fold change) in heart failure vs. controls, indicative of significant molecular perturbation [11]. Strikingly, many upregulated proteins related to the intrarenal RAAS and fibrosis. For instance, angiotensin-converting enzyme (ACE) was >20-fold up, with a 5-fold increase in intrarenal angiotensin II levels. The matricellular protein periostin (POSTN) was ~7-fold up; the receptor for advanced glycation end-products (RAGE) was ~14-fold up. Markers of inflammatory extracellular matrix (ECM) remodeling were enriched, including collagens (type VI collagen), galectin-3, and fibulin (FHL-1), alongside proteins of endothelial dysfunction (vWF, caveolins) and podocyte integrity (ZO-1, CLIC5) [11]. These changes occurred even though glomerular filtration rate remained ostensibly normal, signifying that chronic heart failure initiates a molecular injury program in the kidneys well before overt uremia or kidney dysfunction. The altered renal proteome points to pro-fibrotic and pro-inflammatory pathways that may perpetuate the cardio-renal cycle. Such findings highlight potential early biomarkers and novel drug targets such as RAGE or periostin for preventing Type 2 CRS progression (Figure 2) [12,13].

**Figure 3:**
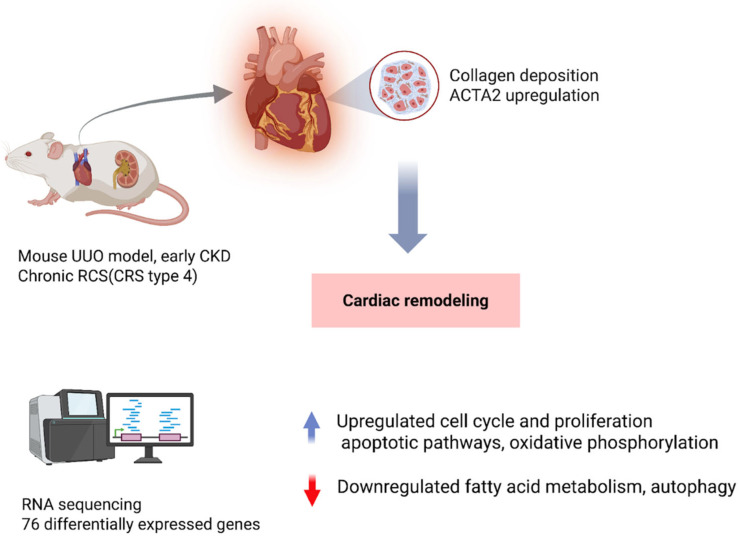
Cardiorenal Syndrome Type 4: Kidney Injury Induces Cardiac Remodeling. Unilateral ureteral obstruction (UUO) in mice, used to model early chronic kidney disease (CKD), triggers cardiac remodeling even in the absence of severe uremia. After 3 weeks, hearts show collagen deposition and increased α-smooth muscle actin (*ACTA2*), with RNA sequencing revealing 76 differentially expressed genes enriched in inflammatory, proliferative, and apoptotic pathways while suppressing mitochondrial metabolism. Larger animal studies corroborate these findings, demonstrating that renal injury drives myocardial transcriptomic reprogramming and progressive CRS within the broader CRS spectrum. Abbreviations: UUO, unilateral ureteral obstruction; CKD, chronic kidney disease; CRS, cardiorenal syndrome; *ACTA2*, actin alpha 2 (smooth muscle actin); Cardiac RNA-seq in UUO mice (GEO: GSE235751).

Conversely, in CRS Type 4), primary kidney disease drives myocardial remodeling and failure [14,15]. Animal studies have shown that even mild or early-stage CKD can induce significant cardiac gene expression changes. For example, Munguia-Galaviz et al. used a mouse model of unilateral ureteral obstruction (UUO) to simulate early CKD and examined cardiac transcriptomics after just 3 weeks of renal injury [16]. The UUO mice developed only low-grade uremia and exhibited signs of cardiac remodeling as shown by collagen deposition and *ACTA2* expression [16]. RNA sequencing of heart tissue identified 76 differentially expressed genes (DEGs) in CKD versus sham hearts. The upregulated genes were enriched in pathways of cell cycle and proliferation, immune/inflammatory responses, cardiac stress repair, and apoptosis, suggesting activation of injury and fibrosis programs in the heart. Concurrently, gene set enrichment analysis showed that key metabolic and organelle function pathways were downregulated; notably, mitochondrial oxidative phosphorylation, fatty acid metabolism, autophagy, and peroxisomal functions were suppressed in the CKD hearts. One interesting transcript was vimentin, a mesenchymal marker that was significantly upregulated in UUO hearts, and this may reflect fibroblast activation or myocyte phenotypic switching. These data emphasize that renal injury can provoke extensive cardiac transcriptional reprogramming characterized by heightened inflammation and fibrosis alongside mitochondrial dysfunction and energy metabolism derangements, all of which likely contribute to the development of heart failure in CRS Type 4. Similarly, in vivo models with more moderate CKD have corroborated these themes. In a translational swine model of non-uremic CKD, Chade et al. demonstrated that chronic kidney damage leading to left ventricular diastolic dysfunction (a CRS Type 4 phenotype) causes marked changes in the cardiac microRNA and mRNA expression profile [17]. After 14 weeks of CKD, pig hearts showed diastolic dysfunction with mild fibrosis and lipid accumulation, and RNA sequencing revealed 125 mRNAs upregulated and 172 downregulated, along with 9 microRNAs up and 17 down (all with ≥2-fold change, FDR ≤ 0.05) compared to controls [17]. Integrated miRNA–mRNA network analysis identified anti-correlated pairs, including 71 downregulated genes that were targets of upregulated miRNAs (and vice versa) [17]. These gene networks pointed to processes such as ubiquitin–proteasome activity, ATP synthesis and fatty acid metabolism, and extracellular matrix remodeling, aligning with the observed cardiac hypertrophy and metabolic impairment. Thus, multi-omics profiling in this large-animal model reinforces that CKD precipitates molecular remodeling of the myocardium, notably a shift toward pro-fibrotic signaling and altered energy utilization, which underlies the clinical cardiac dysfunction. Taken together, animal models across acute and chronic CRS subtypes consistently highlight a convergence on inflammatory, fibrotic, and metabolic gene pathways in affected organs, providing mechanistic clues and validation for therapeutic targets (Figure 3).

## 3. Omics Data from Human Studies

Translational omics investigations in human CRS are more limited, given that CRS is a syndrome spanning multiple conditions and tissue biopsies are not routinely available. Nonetheless, available human data corroborate many findings from experimental models. For example, analysis of public gene expression datasets indicates that certain pro-fibrotic genes are consistently upregulated in both cardiac and renal pathologies relevant to CRS [18,19]. A systems transcriptomic study identified fibronectin-1 (FN1) and periostin (POSTN) as hub genes during CRS progression in rats, and importantly, these were found to be highly expressed across numerous human heart failure and kidney disease datasets as well [20]. In a local cohort of CRS patients vs. controls, FN1 and POSTN levels were significantly elevated and correlated with the degree of cardiac and renal impairment [20]. Notably, each gene demonstrated good diagnostic performance (AUC ~0.77–0.81) in distinguishing CRS patients [20]. When used together in logistic regression and machine learning models, FN1 and POSTN improved the classification of CRS, suggesting these extracellular matrix proteins could serve as biomarkers for CRS detection and risk stratification [20]. These human translational findings echo the animal data that pointed to fibrosis-related genes as central in CRS and validate their relevance in actual patients.

Beyond mRNA signatures, circulating biomarkers and “omics” in blood or urine provide minimally invasive windows into the CRS molecular milieu. Urine peptidomic analysis is one such approach that has been applied to human CRS. In a large cohort study (*n* ≈ 353 CRS patients and matched controls), urine peptide profiling uncovered 559 peptides with significantly altered excretion in CRS, of which 193 peptides appeared to be specific to CRS (differentiating CRS from isolated heart failure or isolated CKD) [3]. Many of these peptides derived from collagen fragments and other extracellular matrix components, again underscoring a strong fibrosis signal in CRS. Using bioinformatic tools, investigators predicted the upstream proteases responsible for the peptide patterns and found enrichment of matrix metalloproteinases (MMP-2, -9, -13), cathepsins, and other proteases that drive tissue remodeling. Indeed, three MMPs alone (MMP-13, MMP-9, MMP-2) accounted for ~44% of the cleavage events generating the CRS-specific urinary peptides. Pathway analysis confirmed that ECM turnover, fibrosis and inflammation were prominent processes reflected in the urinary peptidome of CRS patients. This study provides proof-of-concept that urinary proteomic biomarkers can capture the molecular processes active in both heart and kidney during CRS, potentially enabling earlier diagnosis or monitoring of disease activity by detecting the footprints of organ crosstalk, such as collagen degradation products from ongoing fibrosis.

Inflammatory mediators are another link between cardiac and renal injury in humans [21,22]. For instance, patients with acute CRS (Type 1) have been found to exhibit heightened levels of gut-derived endotoxin (lipopolysaccharide, LPS) and cytokines in circulation, implicating the gut–microbiome axis and systemic inflammation in acute heart–kidney injury [23]. In an observational study including 31 acute heart failure (AHF) patients, 20 CRS-1 patients (AHF complicated by AKI), and 17 healthy controls, plasma LPS concentrations were markedly higher in CRS-1 than in AHF alone (median 118.2 pg/mL [IQR 77.8–217.6] vs. 13.5 pg/mL [12.0–17.0], *p* = 0.008), corresponding to approximately 8-fold elevation [23]. CRS-1 patients also had higher levels of IL-6 (about 5-fold), IL-18 (about 1.5-fold), and the oxidative stress marker MPO (about 1.5-fold) compared with AHF, while TNF-α did not differ significantly. Correlation analyses showed that LPS strongly correlated with IL-6 (r = 0.79), IL-18 (r = 0.77), and MPO (r = 0.80) (all *p* < 0.001). These findings support the concept that acute cardiac decompensation can cause gut congestion, ischemia, and bacterial endotoxin translocation, which in turn provoke systemic inflammation and oxidative stress, leading to renal endothelial injury and dysfunction. LPS and its downstream cytokines may therefore serve as mechanistic mediators and potential biomarkers of acute CRS, though confirmation in larger studies is needed.

Overall, human omics and biomarker studies, though in early stages, reinforce the concept that common molecular pathways, especially fibrosis, inflammation, and metabolic dysregulation, may underlie the cardiorenal axis. The convergence of evidence from animal models and human data lends confidence that these pathways are genuine drivers of CRS pathogenesis and not merely experimental artifacts. Still, human-specific genomic studies remain scarce, partly because CRS is a heterogeneous syndrome rather than a single defined disease. This gap highlights the need for further translational research applying multi-omics to patient cohorts, to validate targets identified in animals and perhaps uncover human-specific factors (such as genetic polymorphisms or epigenetic modifications) that modulate an individual’s risk of developing CRS in the setting of heart or kidney disease.

## 4. Molecular Pathways and Gene Activation in CRS

**Figure 4:**
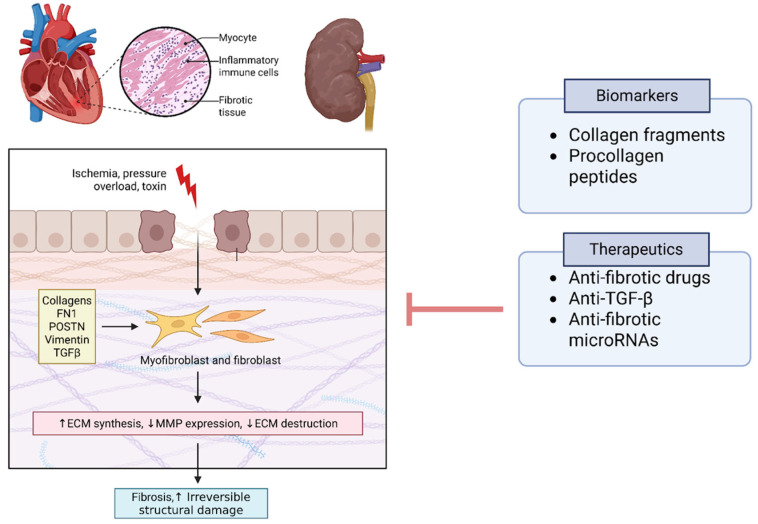
Fibrotic Pathways as a Cornerstone of CRS. Multi-omics studies consistently implicate fibrosis and extracellular matrix (ECM) remodeling as central pathways in CRS pathogenesis. Differentially expressed genes and proteins include fibronectin-1 (FN1), periostin (POSTN), collagens, and cytoskeletal proteins such as vimentin, alongside signaling mediators in the transforming growth factor-β (TGF-β) pathway. Matrix metalloproteinases (MMPs) and related protease inhibitors reflect active ECM turnover and remodeling. Biomarkers of collagen degradation (e.g., urinary fragments, circulating procollagen peptides) may enable early detection of CRS prior to functional decline. Therapeutic targeting of fibrosis through anti-fibrotic drugs, anti-TGF-β strategies, and microRNA modulation is a promising translational direction. Abbreviations: FN1, fibronectin-1; POSTN, periostin; TGFβ, transforming growth factor beta; ECM, extracellular matrix; MMP, matrix metalloproteinase; PRIDE accessions listed in Table S1).

A unifying theme emerging from multi-omics research is that fibrosis and extracellular matrix remodeling constitute a central pathway in CRS. Both cardiac and renal tissues respond to chronic injury with fibrotic remodeling, and CRS appears to amplify this process through organ cross-talk. High-throughput studies consistently find markers of fibrosis among the top differentially expressed genes or proteins: examples include collagens, fibronectin-1 (FN1), periostin (POSTN), vimentin, and transforming growth factor-β (TGF-β) signaling components, all of which have been implicated in the myocardium and kidneys of CRS models [20]. A 2021 proteomics review concluded that fibrosis is likely the “cornerstone” unifying most CRS pathways—various upstream injuries (ischemia, pressure overload, toxin accumulation, etc.) converge on fibrogenesis in both organs, leading to irreversible structural damage [24]. Notably, biomarkers reflecting collagen turnover and ECM degradation (for instance, peptide fragments detected in urine or circulating procollagen peptides) may show promise for early detection of CRS before overt functional decline [24]. Targeting fibrotic pathways whether via anti-fibrotic drugs, anti-TGF-β therapies, or novel anti-fibrotic microRNAs is a promising area under investigation, supported by omics associations (Figure 4).

Inflammation and immune activation are another key molecular axis in CRS and heart failure pathogenesis [25]. Transcriptomic profiles of both acute and chronic CRS models demonstrate upregulation of cytokines, chemokines, and immune cell recruitment signals in the affected heart and kidney [7,26,27,28]. For example, IL-6 is elevated in acute CRS patients and likely mediates organ injury. Gene sets related to macrophage activation, leukocyte trafficking, and complement cascade are often enriched in CRS tissue profiles [21]. In chronic settings, the persistent low-grade inflammation associated with heart failure and CKD can synergize, leading to compounded cytokine release and oxidative stress that damages both organs. Oxidative stress markers like myeloperoxidase and NADPH oxidase components are elevated, indicating that redox imbalance accompanies the inflammatory injury [29]. Mechanistic studies suggest that therapies reducing inflammation might attenuate CRS progression, although clinical evidence is still limited [30].

**Figure 5:**
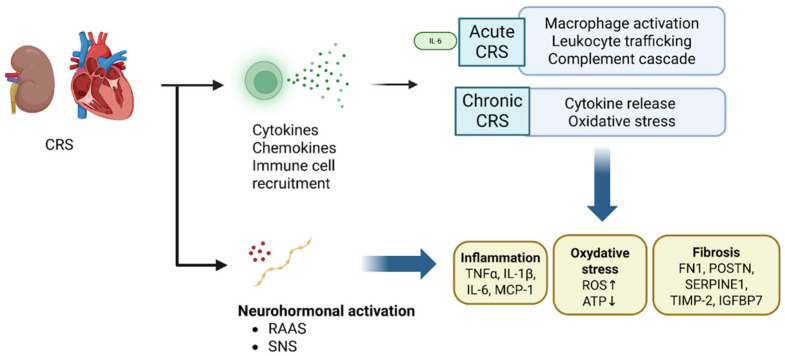
Inflammation, Oxidative Stress, and RAAS Hyperactivation in CRS. Inflammatory and immune pathways are strongly upregulated in CRS, with transcriptomic profiles showing cytokine release, macrophage recruitment, leukocyte trafficking, and complement cascade activation. Oxidative stress markers such as myeloperoxidase and NADPH oxidase components further damage cardiac and renal tissue. Parallel proteomic studies demonstrate marked activation of RAAS, with renal ACE levels >20-fold higher and angiotensin II massively elevated, promoting vasoconstriction, fibrosis, and inflammatory signaling through AT1 receptors. Aldosterone excess exacerbates myocardial fibrosis and renal injury. These pathways intersect, as angiotensin II can stimulate TGFβ and ROS production, linking neurohormonal, inflammatory, and fibrotic mechanisms in CRS progression. Abbreviations: CRS, cardiorenal syndrome; RAAS, renin–angiotensin–aldosterone system; SNS, sympathetic nervous system; IL-6, interleukin-6; IL-1, interleukin-1; MCP-1, monocyte chemoattractant protein-1; TNFα, tumor necrosis factor alpha; ROS, reactive oxygen species; ATP, adenosine triphosphate;FN1, fibronectin-1; POSTN, periostin; SERPINE1, serpin family E member 1; TIMP-2, tissue inhibitor of metalloproteinases-2; IGFBP7, insulin-like growth factor-binding protein 7.

Another recurring molecular player is the RAAS and related pathways of neurohormonal activation. RAAS hyperactivity is well known in both heart failure and kidney disease independently; omics studies confirm that RAAS components are upregulated in CRS and may be disproportionately activated when both organs are failing. The proteomic finding of a >20-fold increase in renal ACE and massively elevated intrarenal angiotensin II in a heart failure model is telling [11]. Angiotensin II not only causes vasoconstriction and hemodynamic stress but also directly stimulates fibrosis and inflammatory gene expression via AT1 receptors in heart and kidney tissues [31,32]. Likewise, aldosterone excess contributes to myocardial fibrosis and renal injury, hence the benefit of mineralocorticoid receptor blockers in cardiorenal patients. RAAS interacts with other molecular pathways; for example, angiotensin II can induce TGF-β (fibrosis) and reactive oxygen species production (oxidative stress), linking these major CRS mechanisms. The genetic predisposition of RAAS pathway activity (e.g., polymorphisms in the ACE gene or angiotensinogen) in CRS is an area of interest, though not yet fully elucidated; conceivably, patients with high baseline RAAS activity might be at greater risk of combined heart–kidney decline (Figure 5) [33].

Metabolic dysregulation and mitochondrial dysfunction have come to the forefront as well, particularly in chronic CRS. The heart in patients with CKD often exhibits energy starvation, shifts in substrate utilization, and mitochondrial abnormalities (a phenomenon sometimes termed “uremic cardiomyopathy”). Transcriptomics from CKD models show downregulation of genes for fatty acid oxidation, electron transport chain subunits, and peroxisome proliferator-activated receptor (PPAR) signaling in cardiac tissue [16]. One mechanistic link is the elevation in circulating uremic toxins such as p-Cresyl sulfate in CKD, which can promote apoptosis in cardiomyocytes [34]. Similarly, anemia of CKD and iron dysregulation can worsen myocardial energy deficits. In the kidney, longstanding heart failure can lead to congestion and reduced renal oxygen delivery, favoring a switch to anaerobic metabolism and fibrosis. Restoration of mitochondrial function and metabolic flexibility (for instance, via PPAR agonists or novel metabolism-targeted drugs) might therefore hold therapeutic value in CRS Type 4; intriguingly, one study in a mouse CRS model identified PPAR-α downregulation in the heart as a critical mediator of dysfunction and showed that microRNA-21 (a known inhibitor of PPAR-α) was upregulated, linking a metabolic deficit to a regulatory microRNA that could be targeted [35,36].

**Figure 6:**
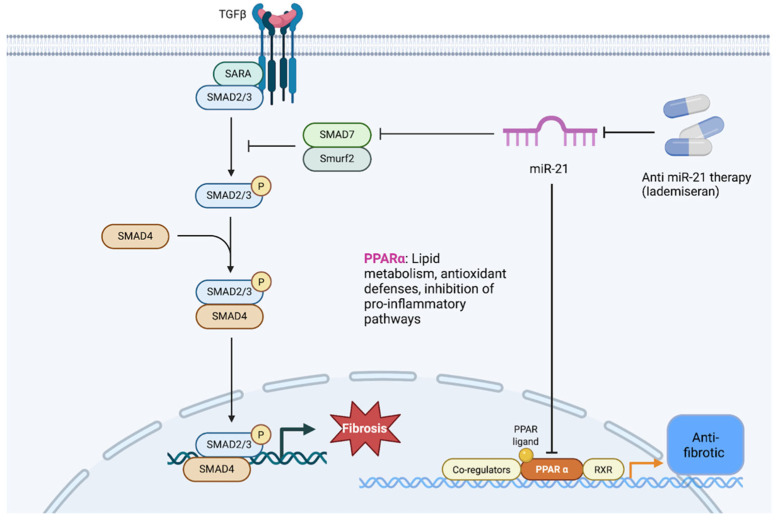
Mechanistic role of miR-21 in fibrosis. miR-21 is upregulated in cardiac and renal injury and represses PPAR-α, a metabolic and anti-fibrotic regulator. Loss of PPAR-α removes inhibitory control over oxidative stress and TGF-β signaling. In parallel, miR-21 suppresses SMAD7, thereby enhancing TGF-β/SMAD2/3/4 signaling. This cascade drives fibroblast activation, myofibroblast transition, extracellular matrix deposition, and progressive fibrosis. Antisense oligonucleotide therapy against miR-21 (e.g., lademirsen) has shown efficacy in preclinical models of kidney and heart fibrosis, although early clinical trials revealed limited benefit, underscoring the complexity of targeting a single microRNA in CRS. Abbreviations: PPAR-α, peroxisome proliferator-activated receptor-α; TGF-β, transforming growth factor-β; SMAD, homologs of Drosophila mothers against decapentaplegic; ECM, extracellular matrix; CRS, cardiorenal syndrome.

Finally, epigenetic modifications and non-coding RNAs are increasingly recognized as modulators of the above pathways in CRS. Epigenetics refers to heritable changes in gene expression not due to DNA sequence variation including DNA methylation, histone modifications, and regulatory RNAs. Both heart and kidney diseases have been associated with epigenetic alterations (e.g., aberrant DNA methylation in fibrotic genes, histone acetylation changes driving hypertrophy) [27]. However, dedicated studies of epigenetics in cardiorenal syndrome per se are still nascent. Given the dynamic and multi-factorial nature of CRS, epigenetic mechanisms could serve as a common soil that primes both organs to injury. For example, chronic inflammation and oxidative stress in CRS may lead to DNA hypermethylation of anti-fibrotic genes or acetylation of histones that upregulate pro-inflammatory genes, thus perpetuating organ damage. Small non-coding RNAs, particularly microRNAs (miRNAs), have emerged as important post-transcriptional regulators in CRS. Several miRNAs are dysregulated during heart or kidney failure and some appear to influence both organs. A prime example is miR-21, which is consistently upregulated in models of cardiac fibrosis and renal fibrosis [5]. MiR-21 promotes pro-fibrotic signaling, possibly through miR-21-5p–mediated translational repression of PPAR-α and activation of TGF-β/SMAD signaling, leading to fibroblast activation and extracellular matrix deposition (Figure 6). Antisense oligonucleotide therapy against miR-21 has shown beneficial results in preclinical models, reducing kidney and heart fibrosis and improving function [37,38]. Translationally, lademiseran (anti-miR-21 therapy) was evaluated in patients with Alport syndrome (NCT03373786), while it demonstrated safety, efficacy signals were limited, underscoring the complexity of targeting single regulatory microRNAs in human disease [39]. Because miR-21 is conserved across species and broadly expressed in vascular, cardiac, and renal cells, it may be considered a universal “cardiorenal” microRNA contributing to fibrogenesis in both organs. In fact, antisense oligonucleotide therapy against miR-21 has shown beneficial results in preclinical models, reducing kidney and heart fibrosis and improving function [40,41]. Other microRNAs such as miR-210, miR-30, miR-146a have been reported in the context of heart failure or CKD [42,43]. These regulators may coordinate the heart–kidney responses to stress, for instance, by being carried in extracellular vesicles from one organ to the other as molecular messengers [7]. Going forward, mapping the epigenetic and non-coding RNA landscape in CRS could reveal higher-order control points that simultaneously affect cardiovascular and renal gene programs.

In summary, multi-omics studies highlight several interlocking molecular axes in CRS: fibrosis or ECM remodeling, inflammation and immune activation, neurohormonal stimulation, oxidative stress, metabolic dysfunction, and epigenetic regulation. Rather than acting in isolation, these pathways form a network of feed-forward loops. For example, RAAS activation leads to fibrosis and oxidative injury; fibrosis causes further inflammation; mitochondrial dysfunction exacerbates oxidative stress, and so on. A visualization of CRS pathophysiology would show the heart and kidney each undergoing these changes and exchanging damaging signals (cytokines, hormones, uremic toxins, microRNAs) that reinforce each other’s injury. Recognizing the common gene expression signatures and pathway activations in both organs provides a molecular basis for why heart and kidney failure so often coexist and worsen together.

## 5. Additional Cardiorenal Connectors

In patients with CKD or when glomerular filtration drops significantly to impair normal phosphate excretion, hyperphosphatemia develops and is thought to be a driving force for the development of adverse cardiovascular outcomes in persons with CKD [44]. As renal dysfunction progresses, there is an inability to excrete dietary phosphorus. The FGF23/Klotho axis is a predominantly bone-derived response to elevated serum phosphorus, which increases renal phosphate excretion [44]. The binding of FGF23 to its primary receptor, FGFR1, is dependent on Klotho. Klotho expressions are found in various tissues, including the liver, pancreas, kidneys, and vasculature [45]. Renal expression of Klotho is found in the proximal and distal convoluted tubules. Several animal models of Klotho deficiency demonstrate increased vascular calcification and aging. CKD is a state of Klotho deficiency and an imbalance in the FGF23/Klotho axis [46]. In tissues that do not express Klotho, such as the myocardium, FGF23 binds to FGFR4 and results in inflammation, fibrosis, and cardiac hypertrophy, which is a hallmark of CRS [47,48]. FGF23 can be expressed independently of phosphorus, and cardiomyocytes can express FGF23 under stress [49].

Several studies have demonstrated that FGF23 serves as a cardiorenal connector, involved in organ crosstalk in animal models of cardiorenal syndromes [48,50,51,52]. Evidence has shown that some of these connections are independent of serum phosphate level, the traditional stimulus of FGF 23 expression. Faul et al. demonstrated that administering FGF23 to isolated rat myocardiocytes resulted in abnormal hypertrophy, which was mediated by FGF23-FGFR calcineurin-NFAT signaling [48]. Intramyocardial and intravenous administration resulted in LVH in both wild-type and Klotho-deficient mice [48]. In addition, a rat model of CKD type 4 CRS induced by 5/6 nephrectomy, these animals develop elevated FGF23, hypertension, and LVH. Faul et al. demonstrated that LVH associated with 5/6 nephrectomy can be attenuated by administration of an FGFR blocker, PD173074 [48].

In a murine model of CRS type 2 induced by surgical myocardial infarction, Hao et al. demonstrated an increase in circulating FGF23 at 12 weeks, as well as upregulation of genes for FGF23 and FGFR4 in cardiac tissue and an upregulation of FGFR4 genes in renal tissue [52]. Through a series of experiments, these investigators demonstrated in this model of murine type 2 CRS that cardiac overexpression of FGF23 induced the upregulation of collagen I, vimentin, TGF-β, p-GSK-3β, and active β-catenin, resulting in both cardiac and renal fibrosis [51,52]. In a murine model of pressure overload CHF induced by transaortic constriction, gene expression of FGF23 and protein expression were upregulated, resulting in myocardial fibrosis and hypertrophy [51]. In murine models of CHF caused by myocardial infarction or ischemia–reperfusion, it was demonstrated that FGF23 exacerbated diastolic dysfunction. In these models, the mechanism driving FGF23-induced myocardial fibrosis and diastolic dysfunction is mediated by upregulation of genes coding for ß-catenin, transforming growth factor β (TGF-β), procollagen I, and procollagen III [51]. In a 5/6 nephrectomy rat model of type 4 CRS, elevated FGF23 levels increase the expression of genes associated with RAAS activation, promoting LVH and fibrosis. The degree of RAAS gene activation correlated with the severity of fibrosis and hypertrophy [50].

In Type 3 CRS, FGF23 can be elevated by classic physiologic drivers such as acute hyperphosphatemia. FGF23 increases the activity of Na-Cl cotransporters at the distal tubule, resulting in avid sodium reabsorption and potential for volume overload [53]. Andrukhova et al. demonstrated that in wild-type mice, administration of FGF23 resulted in hypertension, volume overload, and LVH [53].

Notably, patients who develop AKI are at risk for the development of myocardiocyte necrosis, apoptosis abnormalities in intracellular calcium handling, inflammation, resulting in the development of pathologic myocardiocyte hypertrophy, LVH, and fibrosis [54]. Gonzalez-Lafuente et al., in a murine model of AKI induced by Folic Acid, AKI mice developed elevated FGF23 levels, disordered contractile function, a systolic sarcoplasmic Calcium leak, and an LVH phenotype [55]. The LVH phenotype and intracellular calcium abnormalities were abrogated in AKI Transgenic mice overexpressing Klotho [55].

The basic science leading to the understanding of FGF23 as a cardiorenal connector has led to clinical applications, as demonstrated by Ter Matten et al., who showed that patients with congestive heart failure and elevated levels of FGF23 were volume overloaded, not optimally treated, and had poor outcomes [56]. Elevated FGF23 levels have also been found to be predictive of the severity of AKI in infants undergoing cardiac surgery and in critically ill obstetric cases [57].

## 6. Limitations and Future Directions

While emerging omics data have advanced our understanding of CRS, several important limitations must be acknowledged. First, heterogeneity in the definitions and classification of CRS (Types 1–5) across studies complicates direct comparisons and may partly account for variability in reported findings. Second, methodological challenges remain in the application of omics technologies. Discordance between tissue-based analyses and liquid-biopsy assays, as well as potential pre-analytic batch effects in proteomic and miRNA workflows, can introduce variability and limit reproducibility. Moreover, much of the available evidence is associative rather than interventional, which restricts the ability to draw firm conclusions regarding causality.

Another limitation is the reliance on experimental models. Although animal and in vitro systems provide mechanistic insights, they cannot fully replicate human CRS pathophysiology. Rodent models may not capture the influence of comorbidities (diabetes, atherosclerosis) or the prolonged timescales of human disease, and different model systems (surgical, ischemic, or toxin-induced) emphasize distinct pathways, making it difficult to generalize molecular signatures across CRS types [58]. Human studies, on the other hand, are often cross-sectional, with heterogeneity in patient populations, underlying disease etiologies, and medication use that can confound results. Tissue availability further limits human omics studies, as biopsy samples are rarely obtained outside of transplant or autopsy, forcing reliance on blood or urine as proxies that may not fully reflect organ-specific processes.

Finally, most studies have examined a single “omics” layer at a time. Yet, changes in mRNA do not necessarily translate to protein alterations, and metabolic outcomes are often shaped by post-translational regulation [59]. True integrative multi-omics approaches, linking transcriptomic, proteomic, and metabolomic data within the same models or patient cohorts, are still in their infancy. Overcoming these challenges will require standardized CRS definitions, harmonized analytic pipelines, larger well-phenotyped cohorts, and longitudinal profiling to distinguish predictive markers from downstream consequences.

Despite these limitations, the trajectory of CRS research is clearly shifting toward systems-level and translational approaches. Future studies that integrate multi-omics data and identify convergent pathways, such as the emerging miR-21/FN1/POSTN axis, may provide a scaffold for developing targeted therapies. Ultimately, bridging associative molecular insights with interventional strategies remains a critical next step toward precision medicine in CRS.

## 7. Diagnostic and Therapeutic Implications

The genetic and omics insights into CRS carry several implications for improving clinical management. On the diagnostic front, a move is underway to develop biomarkers that reflect the complex pathophysiology rather than relying solely on traditional measures like serum creatinine or B-type natriuretic peptide (BNP). The discovery of molecules such as FN1 and POSTN as potential circulating markers of CRS is promising—a blood test panel measuring these fibrotic markers could potentially aid in early CRS diagnosis or risk prediction [20]. Similarly, inflammatory markers (IL-6, TNFα, high-sensitivity C-reactive protein) or oxidative stress markers (MPO) might be integrated into risk scores if their association with CRS incidence is validated. The ultimate goal is to detect the onset of organ cross-talk early, before irreversible damage occurs. For example, a heart failure patient who begins to show rising levels of kidney injury markers (such as NGAL, KIM-1, cystatin C) or specific fibrosis biomarkers might be identified as entering CRS and managed more aggressively to protect renal function [60,61]. Urine peptidomics, as demonstrated, could also yield a noninvasive test; a defined peptide signature (pattern of collagen fragments, etc.) might serve as a screening tool for CRS in at-risk patients [3].

Beyond proteins, circulating microRNAs are gaining attention as easily measurable biomarkers that integrate information from multiple organs. For instance, circulating miR-21 was shown to distinguish elderly patients with CRS Type 2 (heart failure with CKD) from those with heart failure alone. Although miR-21 alone had moderate diagnostic accuracy (AUC ~0.75), combining it with conventional renal markers like cystatin C increased the AUC to ~0.90, significantly improving sensitivity and specificity. This suggests that multimarker approaches using combinations of omics-derived markers will likely be most effective for diagnosing CRS and stratifying patient risk. Panels that include markers of cardiac wall stress (e.g., NT-proBNP), renal tubular injury (KIM-1, NGAL), fibrosis (pro-collagen peptides, POSTN), and regulatory molecules (selected miRNAs) could provide a comprehensive molecular fingerprint of CRS activity in a patient. Notably, such markers might also serve prognostic purposes: for example, persistently elevated fibrosis markers might predict worse long-term outcomes or non-response to therapy, enabling clinicians to intensify treatment or consider advanced interventions (ultrafiltration, early transplantation referral, etc.) in high-risk CRS patients.

On the therapeutic side, current management of CRS remains largely supportive and is extrapolated from single-organ failure guidelines (e.g., managing heart failure with diuretics and RAAS blockers, managing CKD with renoprotective measures). The new molecular insights offer avenues for more targeted interventions. One exciting area is the development of RNA-based therapies. As mentioned, anti-miR-21 therapy is one candidate; by inhibiting this master regulator of fibrosis, one might concurrently alleviate cardiac and renal fibrosis. Preclinical trials of an antisense oligonucleotide against miR-21 (for instance, RGM-21 or similar compounds) have shown reduction in collagen deposition [5,62]. Another area is epigenetic therapy. Histone deacetylase (HDAC) inhibitors or DNA methylation modulators could, in theory, reverse maladaptive gene expression patterns in both heart and kidney [63,64]. For example, HDAC inhibitors have shown benefit in deoxycorticosterone acetate (DOCA)-induced hypertensive rats in terms of attenuation of hypertrophy and fibrosis and could be explored in CRS contexts where epigenetic silencing of protective genes occurs [65].

Traditional pharmacotherapy is also being informed by omics findings. The identification of RAAS overactivity in CRS provides further rationale for using RAAS inhibitors (ACE inhibitors, angiotensin receptor blockers, mineralocorticoid receptor antagonists) to break the vicious cycle, though caution is needed as these can acutely worsen renal function. Novel agents like ARNIs (angiotensin receptor–neprilysin inhibitors) might confer dual benefits on the heart and kidney by reducing angiotensin II while enhancing beneficial natriuretic peptides; ongoing trials are examining their impact on renal outcomes in heart failure patients. The prominence of inflammation in CRS suggests that anti-inflammatory therapies could be repurposed for CRS to dampen the injurious cross-talk [66]. Likewise, recognition of metabolic defects in CRS hearts has spurred interest in drugs improving cardiac energetics. For instance, SGLT2 inhibitors, initially developed for diabetes, have demonstrated remarkable ability to reduce heart failure hospitalization and slow CKD progression, effectively mitigating CRS in diabetic and even non-diabetic patients [67,68,69,70]. While their exact mechanisms are multifaceted, likely a combination of osmotic diuresis, improved myocardial metabolism and reduced inflammation, SGLT2 inhibitors exemplify how a single therapy can concurrently benefit both organs [71,72].

Ultimately, the therapeutic implications of a genetic/omics perspective on CRS lie in moving from organ-specific treatment to pathway-specific treatment. As we decode the molecular wiring of heart–kidney interactions, we can aim to disrupt the key drivers, including fibrosis, RAAS, inflammation and oxidative stress that damage both organs. This might mean that future CRS management involves an interdisciplinary, mechanism-based protocol. For example, combining a hemodynamic agent (to unload the heart and kidney) with an anti-fibrotic drug, an anti-inflammatory biologic, and a metabolic modulator—a regimen tailored to a patient’s dominant molecular pathology. Although such precision medicine in CRS is still aspirational, it is increasingly conceivable with the knowledge being generated.

## 8. Conclusions

The landscape of CRS research has evolved from a simplistic cardio-centric or nephro-centric view to a dynamic, network-based understanding of heart–kidney crosstalk. A decade ago, the focus was on managing hemodynamics and fluid balance; today, cutting-edge studies are decoding gene networks and signaling pathways that operate across organ systems. This change in perspective has been driven by the advent of multi-omics technologies and high-throughput biology. We now appreciate that CRS is not merely a clinical coincidence of heart failure and kidney failure, but a syndrome of interconnected molecular pathologies. To fully capture this complexity, an integrative multi-omics approach is paramount. We call for large-scale efforts that simultaneously profile the genome, transcriptome, proteome and metabolome of CRS patients and relevant models. By integrating these layers, researchers can construct a systems map of CRS which could identify master regulators, feedback loops, and points of convergence between cardiac and renal injury pathways. Such an approach can also facilitate the development of predictive models and AI-driven analysis, where patterns in multi-omics data could predict CRS onset or response to therapy with high accuracy

In conclusion, viewing cardiorenal syndrome through the lens of genetics and molecular omics has unraveled novel pathways (fibrosis, immune-metabolic interfaces, epigenetic regulation) that were previously unrecognized in the heart–kidney dialog. These insights not only enrich our pathophysiological understanding but also pave the way for innovative biomarkers and therapeutics that transcend traditional organ-specific care. The future of CRS management will likely be shaped by this integrative approach, one that treats the heart and kidney as a unified organ system, guided by the molecular signatures that herald their dysfunction. By embracing an integrative multi-omics strategy, we can move closer to predictive, preventive, and personalized medicine for patients suffering from the dual burdens of cardiac and renal disease. Only through such a holistic approach can we hope to break the intertwined spiral of heart and kidney failure that defines cardiorenal syndrome.

## Data Availability

No new data were created or analyzed in this study.

## Supplementary Materials

The following supporting information can be downloaded at: https://www.mdpi.com/article/10.3390/genes16111303/s1, Table S1: A full dataset index with accession numbers, repositories, and references [73,74].
