# Supplementary material for: Genomics and Multi-Omics Perspectives on the Pathogenesis of Cardiorenal Syndrome
Source: Genes (Basel). 2025 Nov 1;16(11):1303. doi: 10.3390/genes16111303 (PMC12652397; doi:10.3390/genes16111303)
Supplement: Supplementary file 1 [file genes-16-01303-s001.zip › genes-3877044-supplementary.pdf]

Table S1: A full dataset index with accession numbers, repositories, and references

**Figure**	**Model / Tissue**	**Modality**	**Dataset**	**Repository / Platform**	**Reference**
Fig 1	Mouse cardiac arrest/resuscitation → Kidney	Single-nucleus RNA-seq	GEO: GSE271437	GEO (10x Genomics, Illumina)	Burfeind et al. 2025 [9]
Fig 2	Rat aorto-caval fistula (ACF) heart failure → Kidney	Quantitative proteomics (iTRAQ, LC-MS Orbitrap Fusion)	PRIDE: PXD009296	ProteomeXchange / PRIDE	Melenovský et al. 2018 [11]
Fig 3	Mouse UUO (CKD) → Heart	RNA-seq	GEO: GSE235751	GEO (Illumina NovaSeq 6000)	Munguia-Galaviz et al. 2024 [16]
Fig 4	Multi-study (CKD–HF, CKD–CAD, CRS)	Multi-omics integration (urinary & plasma proteomics, CE-MS classifiers)	CKD273 urinary proteome panel; CE-MS plasma proteomics (Schiffer et al. 2011 [73]; Farmakis et al. 2016 [74])	PRIDE / ProteomeXchange (IDs listed in review)	He et al. 2021 [24]
